# Motion Control of a Two-Degree-of-Freedom Linear Resonant Actuator without a Mechanical Spring

**DOI:** 10.3390/s20071954

**Published:** 2020-03-31

**Authors:** Gyunam Kim, Katsuhiro Hirata

**Affiliations:** Department of Adaptive Machine Systems, Graduate School of Engineering, Osaka University, 2-1 Yamadaoka, Suita, Osaka 565-0871, Japan; k-hirata@ams.eng.osaka-u.ac.jp

**Keywords:** two-degree-of-freedom, linear resonant actuator, detent, spring-less, sensor-less, motion control

## Abstract

This study aims to present a new two-degree-of-freedom (DOF) linear resonant actuator (LRA) and its motion control method without a position sensor. The design method of 2-DOF LRA which resonates with only detent force without a mechanical spring is proposed. Since the information of displacement and direction is required to control 2-DOF LRA, a sensor or an estimator is needed. Therefore, we proposed a position estimator and a motion controller for 2-DOF LRA. This paper proved that reciprocating motion, elliptical motion, and scrolling motion can be controlled without a position sensor. Finite element analysis (FEA) and dynamic simulation results validated the proposed method as well.

## 1. Introduction

Recently, new actuators are being actively studied for application to various devices in household appliances and industrial machinery. Linear actuators (LAs) have many benefits due to the fact that they require no mechanical conversion to actuate the mover. Especially, linear resonant actuators (LRAs) offer a gain in efficiency over general linear actuators because they utilize full m–k resonance. Since LRAs can offer high efficiency in linear operation applications, it is mainly used in linear compressors, electric shavers, electric toothbrushes, and so on [1,2,3,4,5]. Meanwhile, multiple- degree-of-freedom (M-DOF) LRA which extends the DOF of LRA has also been studied. M-DOF LRA can be used for applications requiring various motions such as a haptic device [6,7,8,9]. However, the M-DOF LRA requires several mechanical springs for operation, which increases the size and complexity of the actuator. In addition, since the M-DOF LRA also has unrestrained characteristics of the mover stroke, the stroke must be sensed or estimated. In LRAs, the position information of the mover can be obtained by sensors such as linear variable differential transformer (LVDT) and search coil [10]. However, since the sensored method has problems related to cost, assembly tolerance and operating environment, sensorless methods of M-DOF LRAs are needed. LRAs have a disadvantage, which increases complexity in the operation of LRA due to the unconstrained characteristic of the mover stroke (peak-to-peak of displacement). In other words, since the stroke of the LRA may vary depending on the input voltage, input frequency, and load conditions, it is necessary to estimate the stroke. Methods of estimating the stroke by integrating the back electromotive force (EMF) from the motor parameter information and the voltage equation have been proposed [11,12,13,14]. These methods use motor parameters such as coil inductance, resistance, and back-EMF constant. Motor parameters are generally used as a constant obtained from the static state, but the actual value can be changed by the magnet position and the total flux. Therefore, motor parameters of each magnet position and current are stored as a lookup table, and a method of using it for stroke calculation has been proposed [15,16]. However, it has difficulties in measuring motor parameters of all conditions. On the other hand, a method for estimating the stroke by detecting back-EMF in a non-conduction section where the current becomes zero has been proposed [17]. Since this method cannot apply sinusoidal current, the current must include not only the fundamental current component, but also odd harmonic components such as the third and fifth order. As a result, the efficiency of the actuator may be reduced due to the increased current.

In this study, the authors propose an axial gap type 2-DOF LRA with three-phase coils and magnets. This LRA makes it possible to resonate without a mechanical spring. The detent force is designed to generate the force in the center direction wherever the mover is and acts as the stiffness of the spring for resonant operation. Moreover, the current force from three-phase coil can offer a thrust force vector in various directions. Furthermore, we also proposed a method to estimate the position without a sensor. It is confirmed that various motions can be controlled by using the estimated information. Finally, finite element analysis (FEA) is performed to confirm the parameter characteristics of the proposed M-DOF LRA, and the parameter characteristics are used for simulation. The possibility for various motions is verified by the dynamic simulation in a nonlinear model of 2-DOF LRA.

## 2. Proposed 2-DOF LRA

### 2.1. Structure of 2-DOF LRA

**Figure 1:**
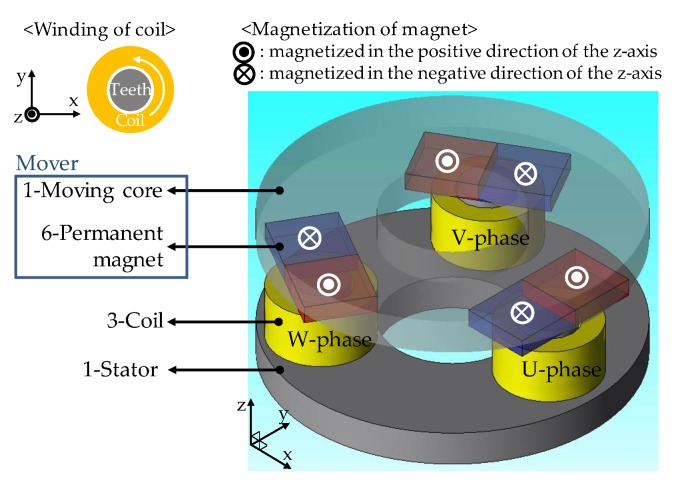
Structure of the proposed two-degree-of-freedom linear resonant actuator (2-DOF LRA).

The structure of the proposed 2-DOF LRA is described in Figure 1. As shown, this actuator consists of stator, moving core, coils, and magnets. Three-phase coils are connected as a star-connection. Magnets N–S arranged on each phase are fixed to the moving core. Although this actuator does not have a mechanical spring, an oscillation with resonant frequency can be performed by using the detent force of the actuator. In addition, since the mover is constrained only in the Z direction, various resonant motions can be operated in the X and Y direction.

**Figure 2:**
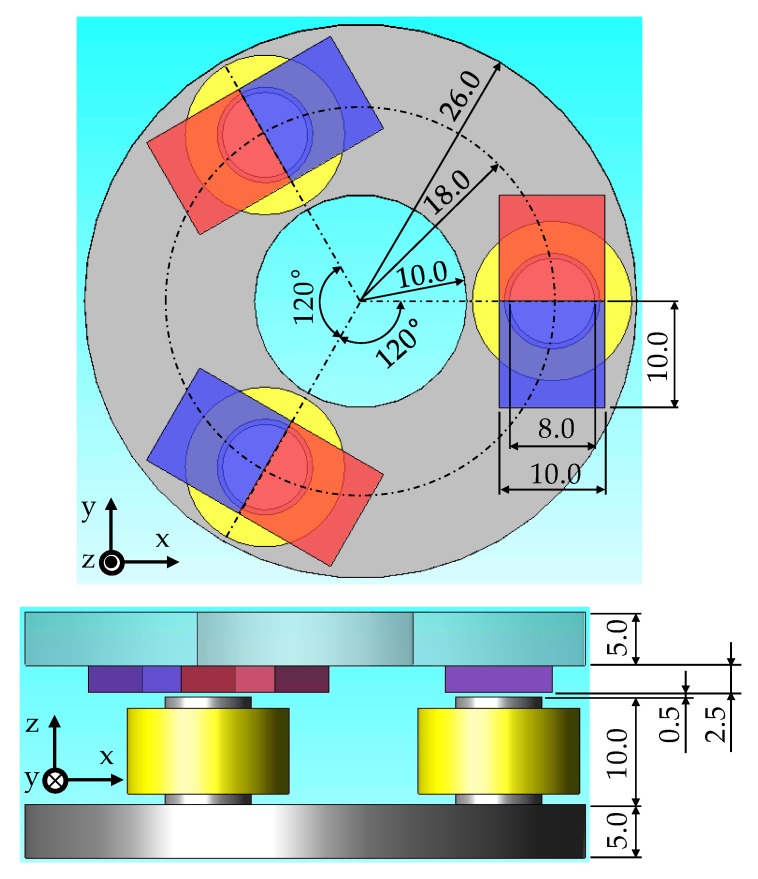
The main dimensions of the proposed 2-DOF LRA.

Figure 2 indicates the main dimensions of the proposed 2-DOF LRA. This actuator has a diameter of 52 mm and a height of 23 mm. The geometric air-gap between the stator and the mover equals 0.5 mm.

### 2.2. Detent Characteristics

**Figure 3:**
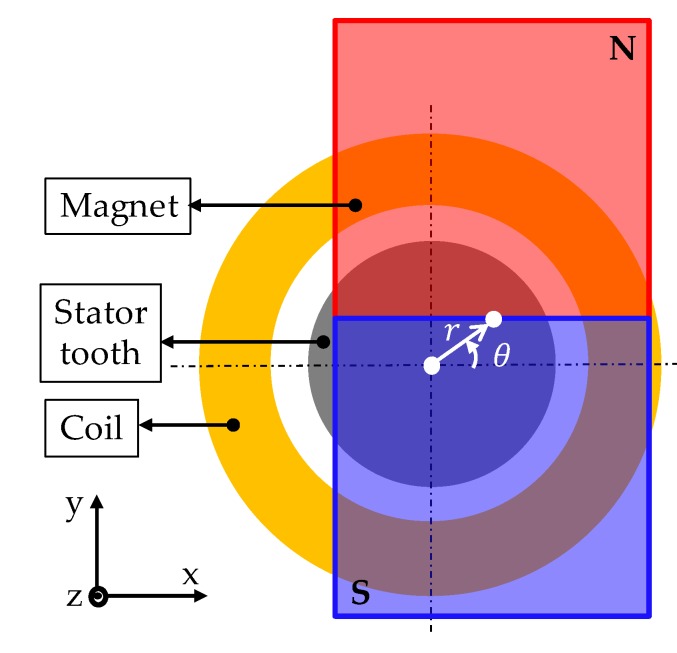
Translation of mover on U-phase.

**Figure 4:**
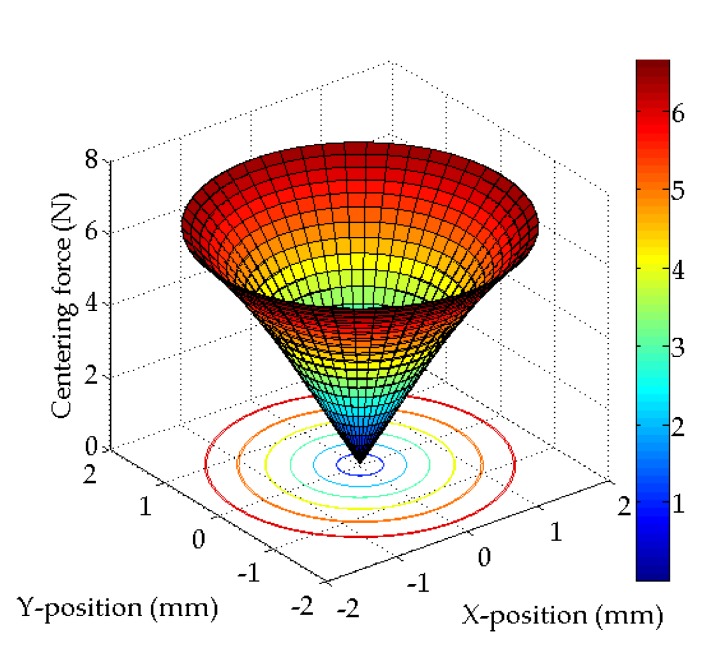
Finite element analysis (FEA) results of centering force (detent force).

Figure 3 shows that the magnet placed on the U-phase is translated in a direction. The magnets of V-phase and W-phase placed by 120/240° are translated in the same direction as U-phase. While the mover is translated by the distance *r* in *θ* direction, the detent force can be defined as follows:

(1) $$F_{dx}=\delta r\left\{-\cos\theta +\frac{1}{2}\cos\left(\theta -\frac{2}{3}\pi \right)+\frac{1}{2}\cos\left(\theta -\frac{4}{3}\pi \right)\right\}$$

(2) $$F_{dy}=\delta r\left\{-\frac{\sqrt{3}}{2}\cos\left(\theta -\frac{2}{3}\pi \right)+\frac{\sqrt{3}}{2}\cos\left(\theta -\frac{4}{3}\pi \right)\right\}$$

(3) $$F_{d\theta }=F_{dx}\cos\theta +F_{dy}\sin\theta =-\frac{3}{2}\delta r$$

where *δ* is the coefficient of slope that represents the relationship between the moving distance of the mover and the force acting on the mover, *F_dx_* is the force in the X-direction, *F_dy_* is the force in the Y-direction, and *F_dθ_* is the force in the *θ*-direction. Equation (3) indicates that the mover always receives centering force in the origin direction wherever it is positioned. This characteristic is similar to the case that several mechanical springs are mounted radially on the X–Y plane. Figure 4 indicates the centering force of 2-DOF LRA obtained by FEA results. As a finite element analysis tool, 3D model transient analysis of JMAG software was used.

### 2.3. Force Constant Characteristics

**Figure 5:**
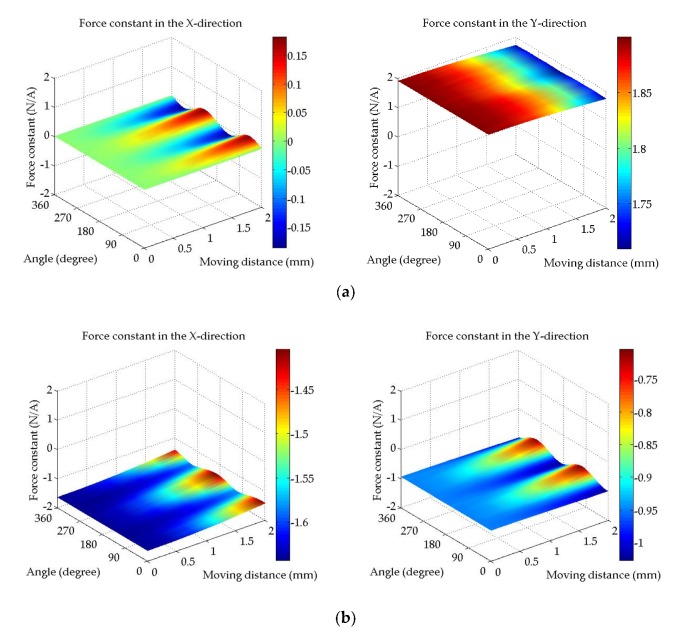

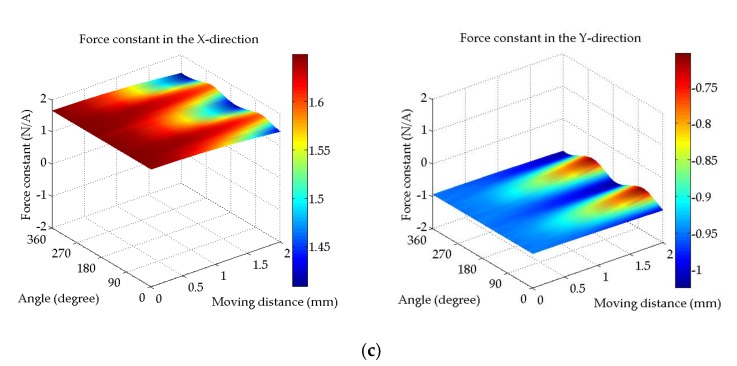
FEA results of force constant (**a**) U-phase, (**b**) V-phase, and (**c**) W-phase.

Figure 5 shows the X and Y components of the force generated by the coil current 1A of each phase in the reference frame. Figure 5a indicates the result of the force constant on the U-phase. As the moving distance *r* increases, it shows non-linear characteristics because the magnet deviates from the tooth. Overall, it can be seen that only the force in the Y-direction is generated. Meanwhile, Figure 5b denotes the result of the force constant on V-phase. Since the V-phase is located at a position rotated 120° from the U-phase, a force constant is generated in a direction rotated 120° from the Y-direction. In the case of Figure 5c, a force constant on W-phase is generated in a direction rotated 240° from the Y-direction.

As the proposed LRA uses a star-connection, assuming that it is no leakage current component, as expressed as follows:

(4) $$i_{u}+i_{v}+i_{w}=0.$$

Furthermore, as the force constant *K_f_* of each phase are all the same, the sum of the torque *T* generated at each phase becomes 0 as follows:

(5) $$T=l\left(F_{u}+F_{v}+F_{w}\right)=lK_{f}\left(i_{u}+i_{v}+i_{w}\right)=0$$

where *l* is the distance between the center of the stator and the center of the tooth, the direction of the force generated on the mover by the current can be defined as follows:

(6) $$F_{ex}=K_{f}\left\{\cos\left(-\frac{1}{2}\pi -\frac{2}{3}\pi \right)i_{v}+\cos\left(-\frac{1}{2}\pi -\frac{4}{3}\pi \right)i_{w}\right\}$$

(7) $$F_{ey}=K_{f}\left\{i_{u}+\cos\left(-\frac{2}{3}\pi \right)i_{v}+\cos\left(-\frac{4}{3}\pi \right)i_{w}\right\}$$

where *F_ex_* is the current force in the X-direction and *F_ey_* is the current force in the Y-direction.

### 2.4. Switch States and Force Direction

**Figure 6:**
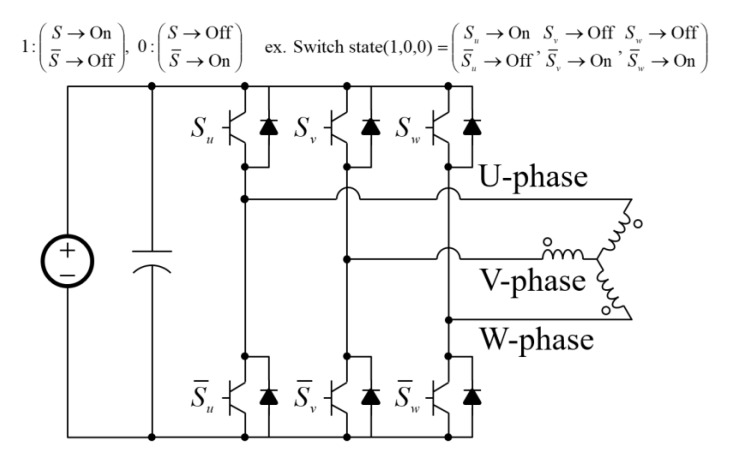
Switch states under three-phase inverter.

Figure 6 illustrates the switching states under a general three-phase voltage source inverter. ‘1’ of the switch state in Figure 6 means that the upper switch is on and the lower switch is off. Conversely, ‘0’ means that the upper switch is off and the lower switch is on. Since there are three legs in the three-phase inverter, a total of 8 switch states exist. However, since the switch states of (1,1,1) and (0,0,0) are defined as zero vectors that cannot apply current to the coil, six effective switch states can be used to generate the force. 

**Figure 7:**
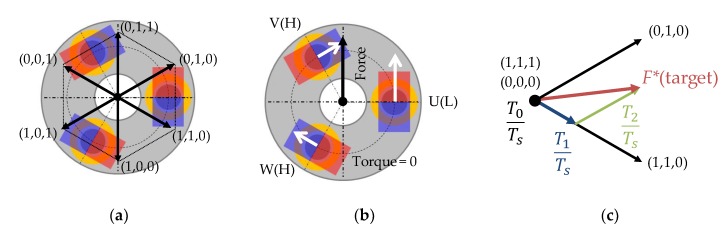
Switch states and force direction: (**a**) force direction in each switch state, (**b**) force direction on (0,1,1), and (**c**) composite of force vector between (1,1,0) and (0,1,0).

Force direction by switch states of three-phase inverter can be generated as shown in Figure 7a. From the current of each phase generated by the switch states and the characteristics of the force constant in Figure 5, a force is applied to the mover in a certain direction. For example, Figure 7b displays that the force direction according to the switch states is determined by the sum of the vector components of the force generated on each phase. 

In addition, the force vector of various directions can be offered by a composite vector of two states as shown in Figure 7c. During the control period *T_s_* of pulse width modulation (PWM), the time *T_0_*, *T_1_*, and *T_2_* of each switch state can be determined to generate a force in the target direction.

## 3. Modelling of 2-DOF LRA

### 3.1. Mechanical Dynamics

The mechanical dynamics of 2-DOF LRA without mechanical spring can be expressed as follows:

(8) $$\left[\begin{array}{c} \dot{x}\\ \ddot{x}\\ \dot{y}\\ \ddot{y} \end{array}\right]=\left[\begin{array}{cccc} 0 & 1 & 0 & 0\\ 0 & -\frac{c}{m} & 0 & 0\\ 0 & 0 & 0 & 1\\ 0 & 0 & 0 & -\frac{c}{m} \end{array}\right]\left[\begin{array}{c} x\\ \dot{x}\\ y\\ \dot{y} \end{array}\right]+\frac{1}{m}\left[\begin{array}{c} 0\\ F_{ex}-F_{dx}-F_{lx}\\ 0\\ F_{ey}-F_{dy}-F_{ly} \end{array}\right]$$

where *x* is the displacement in the X-direction, *y* is the displacement in the Y-direction, *m* is the mass of the mover, *c* is a viscosity coefficient considering the support structure of the mover, *F_ex,ey_* is the force generated by the current, *F_dx,dy_* is the centering force by the detent, and *F_lx,ly_* is the force of load. In addition, the resonant frequency of the 2-DOF LRA can be defined as follows:

(9) $$f_{n}=\frac{1}{2\pi }\sqrt{\frac{k}{m}}=\frac{1}{2\pi }\sqrt{\frac{1}{m}\frac{F_{dx}}{x}}=\frac{1}{2\pi }\sqrt{\frac{1}{m}\frac{F_{dy}}{y}}$$

where *k* is the stiffness of the mechanical spring. However, since the proposed actuator has no mechanical spring, the detent force divided by the moving distance functions as a stiffness.

From the result of the detent force obtained by FEA, the resonant frequency for translational motion is the same regardless of the direction.

### 3.2. Electrical Dynamics

The electrical dynamics of the 2-DOF LRA with three-phase coils can be expressed as follows:

(10) $$\left[\begin{array}{c} v_{u}\\ v_{v}\\ v_{w} \end{array}\right]=\left[\begin{array}{ccc} R_{u} & 0 & 0\\ 0 & R_{v} & 0\\ 0 & 0 & R_{w} \end{array}\right]\left[\begin{array}{c} i_{u}\\ i_{v}\\ i_{w} \end{array}\right]+\frac{d}{dt}\left\{\left[\begin{array}{ccc} L_{uu} & M_{uv} & M_{uw}\\ M_{vu} & L_{vv} & M_{vw}\\ M_{wu} & M_{wv} & L_{ww} \end{array}\right]\left[\begin{array}{c} i_{u}\\ i_{v}\\ i_{w} \end{array}\right]\right\}+\left[\begin{array}{c} e_{u}\\ e_{v}\\ e_{w} \end{array}\right]$$

where *v* and *i* denote the phase voltage and the phase current, *R* is the coil resistance, *L* is the self-inductance, *M* is the mutual inductance, *e* is the back-EMF of each phase. In the case of this LRA, since each phase is arranged symmetrically, it can be satisfied as:

(11) $$R_{u}=R_{v}=R_{w}=R$$

(12) $$L_{uu}=L_{vv}=L_{ww}$$

(13) $$M_{uv}=M_{vw}=M_{wu}=M.$$

Substituting Equations (11)–(13) into Equation (10) can be expressed as follows:

(14) $$\left[\begin{array}{c} v_{u}\\ v_{v}\\ v_{w} \end{array}\right]=\left[\begin{array}{ccc} R+\rho L & 0 & 0\\ 0 & R+\rho L & 0\\ 0 & 0 & R+\rho L \end{array}\right]\left[\begin{array}{c} i_{u}\\ i_{v}\\ i_{w} \end{array}\right]+\left[\begin{array}{c} e_{u}\\ e_{v}\\ e_{w} \end{array}\right]$$

where *ρ* = *d*/*dt*, *L* is a relational expression of leakage inductance, self-inductance, and mutual inductance.

### 3.3. Voltage Reference for Motion Control

**Figure 8:**
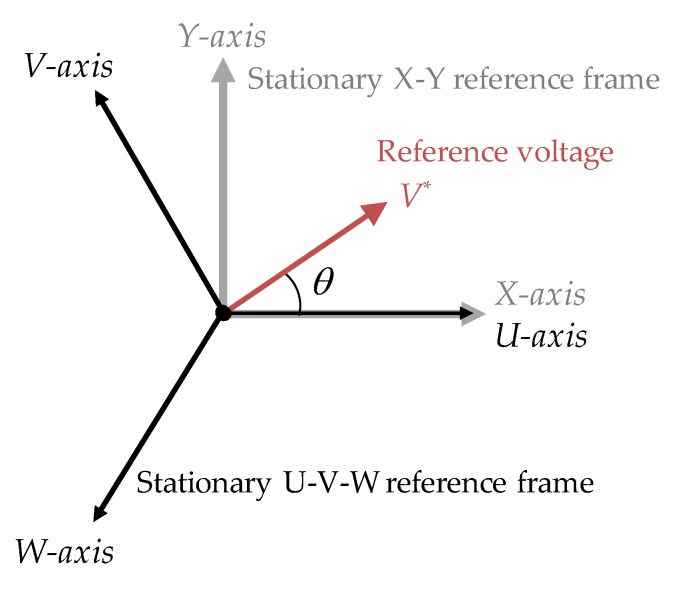
Reference voltage and stationary reference frame.

The vector direction of the force generated by voltage reference in the space vector is rotated by −90°. Figure 8 illustrates the reference voltage in the reference frame. In order to generate voltage in the target direction, voltage must be expressed in the U–V–W reference frame. Therefore, the voltage reference can be transformed in the reference frame as follows:

(15) $$\left[\begin{array}{c} v_{u}\\ v_{v}\\ v_{w} \end{array}\right]=\left[\begin{array}{cc} 1 & 0\\ -\frac{1}{2} & \frac{\sqrt{3}}{2}\\ -\frac{1}{2} & -\frac{\sqrt{3}}{2} \end{array}\right]\left[\begin{array}{c} V^{*}\cos\theta \\ V^{*}\sin\theta \end{array}\right]$$

where V* is a vector component of reference voltage in the reference frame. 

### 3.4. Load Defined

The load acting on the actuator can be defined as a viscous component and a friction component. Therefore, the load acting in the *θ* direction can be defined as follows:

(16) $$F_{l\theta }=F_{l}\mathrm{sgn}\left({\dot{x}}_{\theta }\right)+c_{l}{\dot{x}}_{\theta }$$

where *F_lθ_* denotes the load in the *θ* direction, *F_l_* means the friction load, and *c_l_* stands for coefficient of a viscous load. 

By converting Equation (16) into the XY component, load equation can be obtained as:

(17) $$\left[\begin{array}{c} F_{lx}\\ F_{ly} \end{array}\right]=\left[\begin{array}{c} F_{l}\mathrm{sgn}\left(\dot{x}\cos\theta +\dot{y}\sin\theta \right)\cos\theta +c_{l}\dot{x}\\ F_{l}\mathrm{sgn}\left(\dot{x}\cos\theta +\dot{y}\sin\theta \right)\sin\theta +c_{l}\dot{y} \end{array}\right]$$

(18) $$\theta =\tan^{-1}\left(\frac{\dot{y}}{\dot{x}}\right)$$

where *F_lx_* is the load in the X-direction and *F_ly_* is the load in the Y-direction.

### 3.5. Model Block Diagram

**Figure 9:**
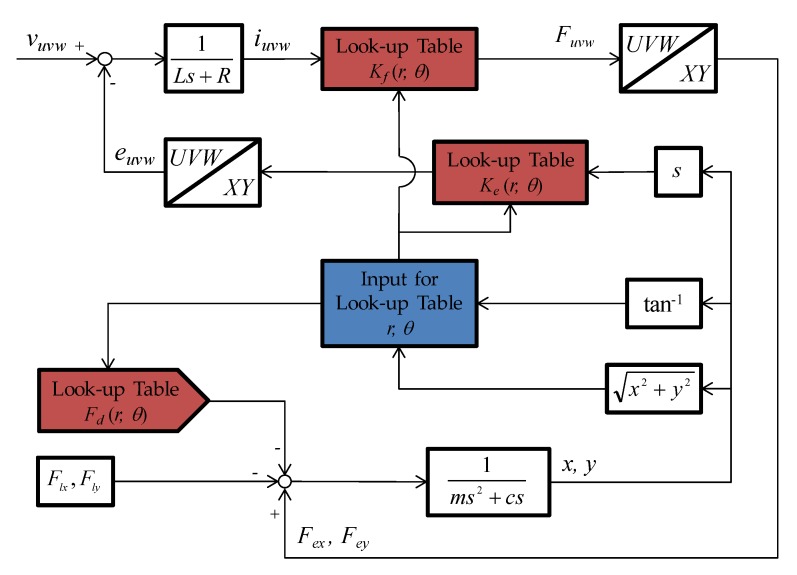
Model block diagram with non-linear parameters.

Figure 9 illustrates the block diagram of 2-DOF LRA. As shown in Figure 9, force constant, back-EMF constant, and detent force are used as the look-up table with FEA results. Inputs of each look-up table are obtained by converting position information (*x*, *y*), which is the output of the model to moving distance (*r*) and angle (*θ*).

## 4. Estimation Method of Motion

**Figure 10:**
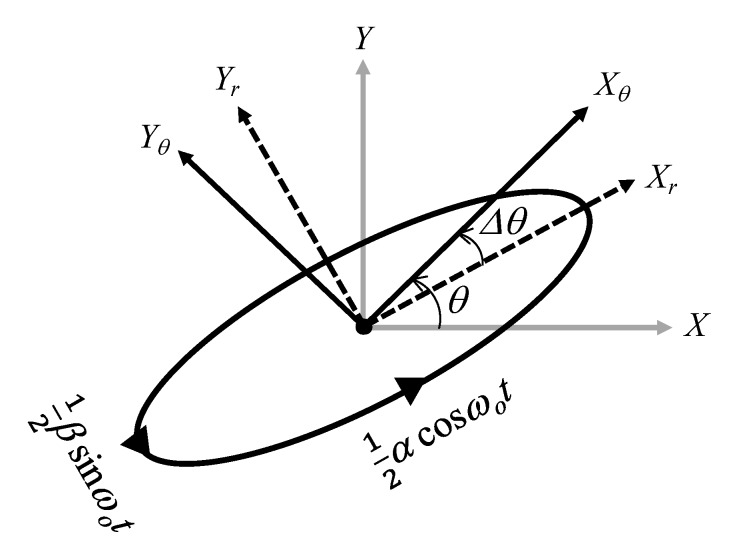
Reference frame of translational motion.

The voltage equation of the 2-DOF LRA in the XY reference frame can be expressed as follows:

(19) $$\left[\begin{array}{c} v_{x}\\ v_{y} \end{array}\right]=\left[\begin{array}{cc} R+\rho L & 0\\ 0 & R+\rho L \end{array}\right]\left[\begin{array}{c} i_{x}\\ i_{y} \end{array}\right]+K_{e}\left[\begin{array}{cc} 0 & 1\\ -1 & 0 \end{array}\right]\left[\begin{array}{c} \dot{x}\\ \dot{y} \end{array}\right]$$

where *K_e_* denotes the back-EMF constant. From Equation (19), position and angle information can be estimated as follows:

(20) $$\left[\begin{array}{c} \hat{x}\\ \hat{y} \end{array}\right]=\frac{1}{K_{e}}\left[\begin{array}{c} \int \left(-v_{y}+Ri_{y}\right)dt+Li_{y}\\ \int \left(v_{x}+Ri_{x}\right)dt-Li_{x} \end{array}\right]$$

(21) $$\left[\begin{array}{c} {\hat{x}}_{\theta }\\ {\hat{y}}_{\theta } \end{array}\right]=\left[\begin{array}{cc} \cos\theta & \sin\theta \\ -\sin\theta & \cos\theta \end{array}\right]\left[\begin{array}{c} \hat{x}\\ \hat{y} \end{array}\right]$$

where *x_θ_* and *y_θ_* represent a position in the orthogonal frame, which are rotated by an angle *θ*. From Equation (21), stroke can be obtained as follows:

(22) $$\left[\begin{array}{c} \hat{\alpha }\\ \hat{\beta } \end{array}\right]=\left[\begin{array}{c} \max\left({\hat{x}}_{\theta }\right)-\min\left({\hat{x}}_{\theta }\right)\\ \max\left({\hat{y}}_{\theta }\right)-\min\left({\hat{y}}_{\theta }\right) \end{array}\right]$$

where *α* and *β* are the stroke position. Since the stroke uses the maximum and minimum values of the displacement, it is updated every half cycle of the operating frequency. Figure 10 shows a type of elliptical translational motion with 2-DOF in the reference frame. If elliptical motion can be controlled, scrolling motion and reciprocating motion can be performed by changing the ratio of *α* and *β*. In Figure 10, *θ* is the reference angle of motion, and Δ*θ* is the error between the reference angle and the actual angle of motion. Therefore, Δ*θ* should be controlled to be zero for more precise motion. The estimated position in the reference frame which is rotated by an angle *θ* can be expressed as follows:

(23) $$\left[\begin{array}{c} x_{\theta }\left(t\right)\\ y_{\theta }\left(t\right) \end{array}\right]=\frac{1}{2}\left[\begin{array}{cc} \cos\Delta \theta & \sin\Delta \theta \\ -\sin\Delta \theta & \cos\Delta \theta \end{array}\right]\left[\begin{array}{c} \alpha \cos\omega_{o}t\\ \beta \sin\omega_{o}t \end{array}\right].$$

Using the orthogonality of *x_θ_*(*t*) and *y_θ_*(*t*) in Equation (23), Δ*θ* can be estimated as follows:

(24) $$\frac{1}{T}\int_{0}^{T}x_{\theta }\left(t\right)y_{\theta }\left(t\right)dt=\frac{\beta^{2}-\alpha^{2}}{16}\sin2\Delta \theta$$

(25) $$\Delta \hat{\theta }≅\frac{8}{\left({\hat{\beta }}^{2}-{\hat{\alpha }}^{2}\right)}\frac{1}{T}\int_{0}^{T}{\hat{x}}_{\theta }\left(t\right){\hat{y}}_{\theta }\left(t\right)dt.$$

where *T* means the time of once driving cycle in a motion.

**Figure 11:**
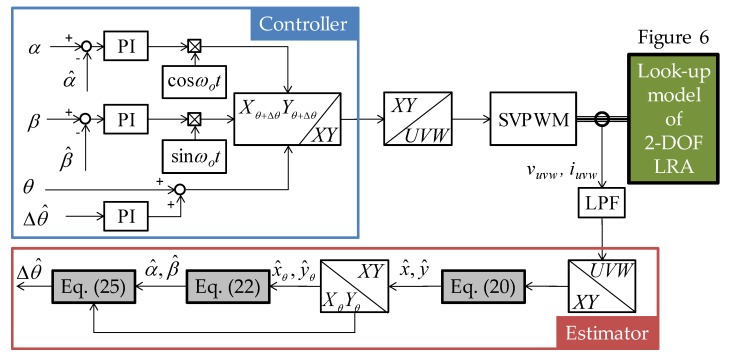
Overall control and estimation block diagram of the proposed 2-DOF LRA.

Figure 11 shows the overall control and estimation block diagram for the dynamic simulation. The PI control is performed by comparing *α*, *β*, and Δ*θ* estimated from Equations (22) and (25). Section 5 details the simulation results using the estimator and controller in Figure 11.

## 5. Simulation Result

The conditions of the dynamic simulation for the verification of the proposed method are shown in Table 1. Force constant, back-EMF constant, and detent force are used as the look-up table with FEA results. In order to simulate a real three-phase inverter, the voltage reference was applied after converted to space vector pulse width modulation (SVPWM).

**Figure 12:**
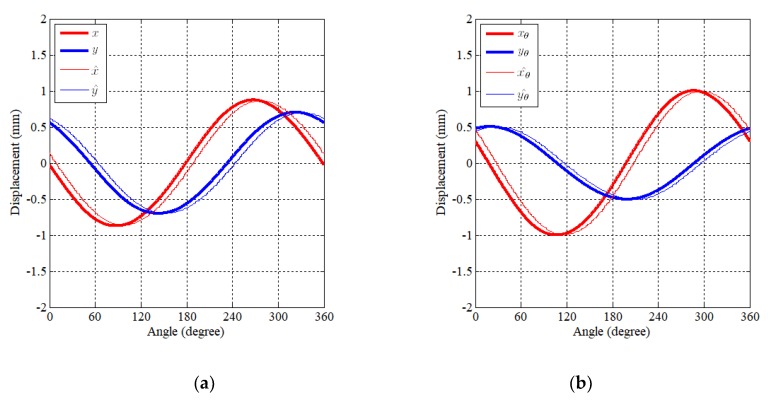
Displacement estimation of elliptical motion (*α*: 1 mm, *β*: 0.5 mm, *θ*: 35 deg.) (**a**) Displacement in the X–Y reference frame and (**b**) displacement in the X_θ_ –Y_θ_ reference frame.

In order to control various motion, the displacement information must be known. Figure 12 presents the estimation result of the displacement information with elliptical motion. Figure 12a indicates displacement information in the stationary reference frame while the result in the rotating reference frame is represented in Figure 12b. There is some phase lag due to the effect of a low pass filter. The cutoff frequency of low pass filter is designed as 20-times the operating frequency.

**Figure 13:**
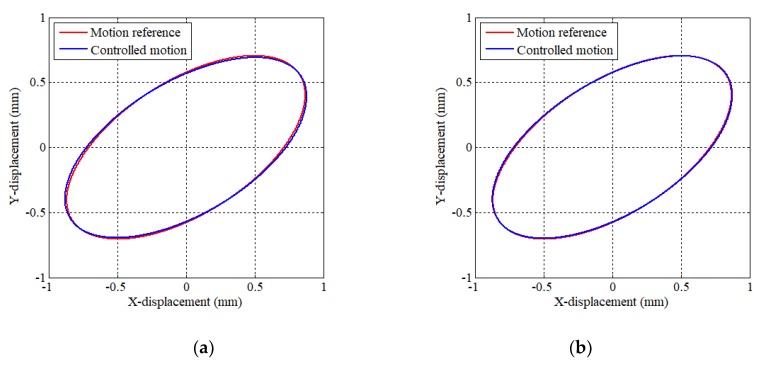
Effect of compensated Δ*θ*. (**a**) without compensation and (**b**) with compensation.

In Section 4, we have mentioned the estimation method of target angle error Δ*θ*. Figure 13a presents the result of elliptical motion without Δ*θ* compensation. On the other hand, Figure 13b shows the case which compensates the estimated Δ*θ*. As a result, precise motion can be realized by compensating the estimated Δ*θ*.

**Figure 14:**
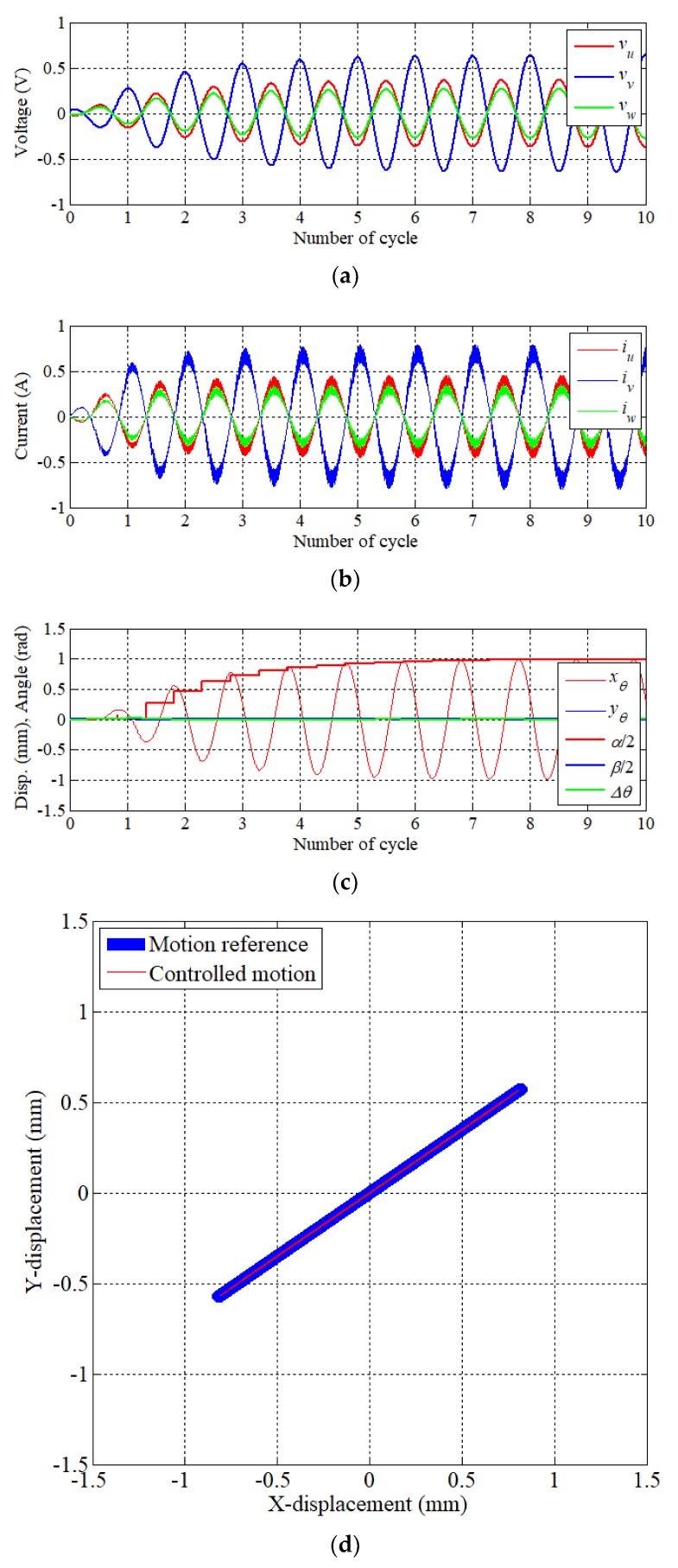
Result of reciprocating motion (*α*: 2 mm, *β*: 0 mm, *θ*: 35 deg.) (**a**) Phase voltage reference, (**b**) phase current, (**c**) displacement, and (**d**) trajectory of motion.

Simulation results of reciprocating motion with a resonant operation are shown in Figure 14. Reference stroke and angle were inputted into the controller as *α* 2 mm, *β* 0 mm, and *θ* 35°. Figure 14a indicates three-phase voltage which represents the fundamental waveform of the PWM. In Figure 14b, three-phase current waveform is illustrated. Each phase current contains some ripple component due to SVPWM. The reciprocating motion can be realized by two-phase currents with the same phase and one phase current with the phase difference of 180°. The amplitude and phase of each phase current are determined by the stroke and direction of the reciprocating motion. Figure 14c indicates the result of displacement (*x_θ_*, *y_θ_*), stroke (*α*, *β*), and angle error (Δ*θ*). It was confirmed that the displacement and stroke in the rotating frame converge into *α* within a period of six cycles, while *β* keeps being 0. Figure 14d presents the trajectory of the displacement on the X–Y plane. As a result, the precise resonance operation can be obtained as the reciprocating trajectory with an angle. 

**Figure 15:**
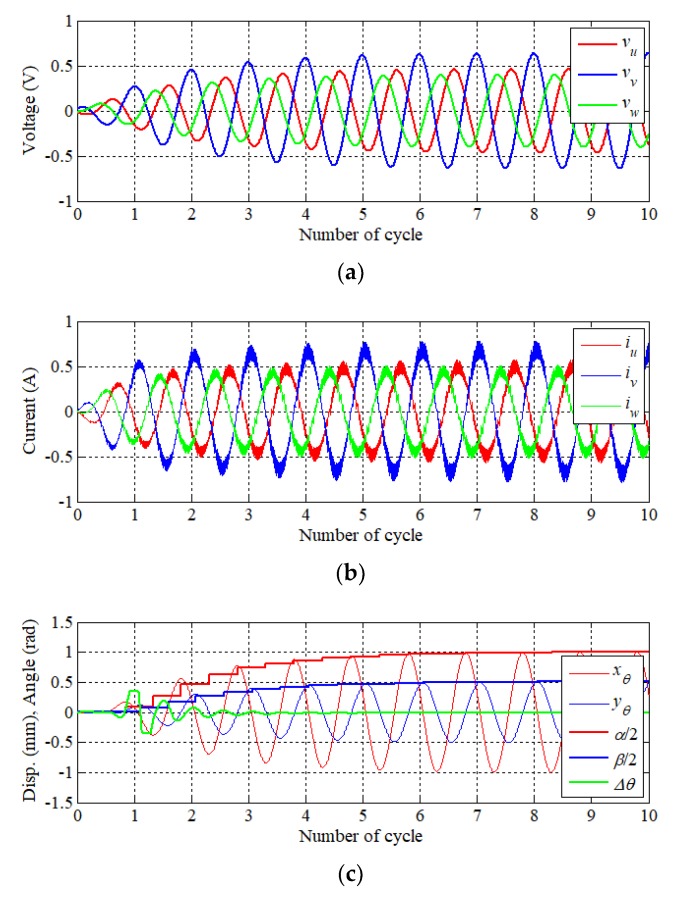

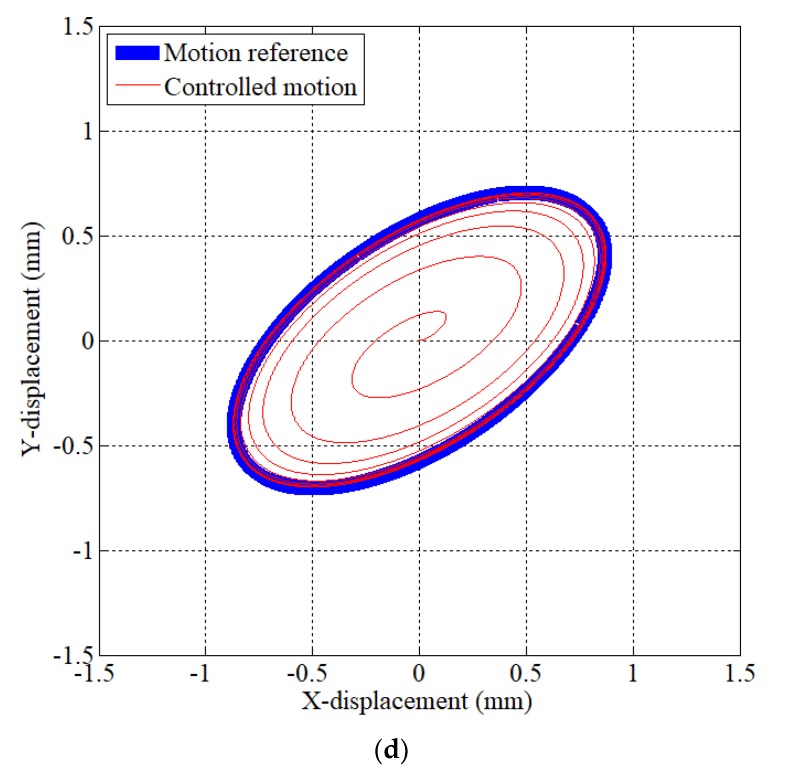
Result of elliptical motion (*α*: 2 mm, *β*: 1 mm, *θ*: 35 deg.) (**a**) Phase voltage reference, (**b**) phase current, (**c**) displacement, and (**d**) trajectory of motion.

Figure 15 illustrates simulation results of elliptical motion. Reference stroke and angle were inputted into the controller as *α* 2 mm, *β* 1 mm, and *θ* 35°. In the case of three-phase permanent magnet synchronous motor (PMSM), each phase current has the same magnitude at a constant load and has a phase difference of 120°. However, it can be seen that the amplitude and phase difference of each phase are changed for the resonance operation of the elliptical motion. A displacement and amplitude at the rotating frame is shown in Figure 15c. It was confirmed that the target motion converges within a period of six cycles from the initial position. The trajectory of the displacement on the X–Y plane is shown in Figure 15d. As a result, it can be seen that resonance operation can be performed as the elliptical trajectory.

**Figure 16:**
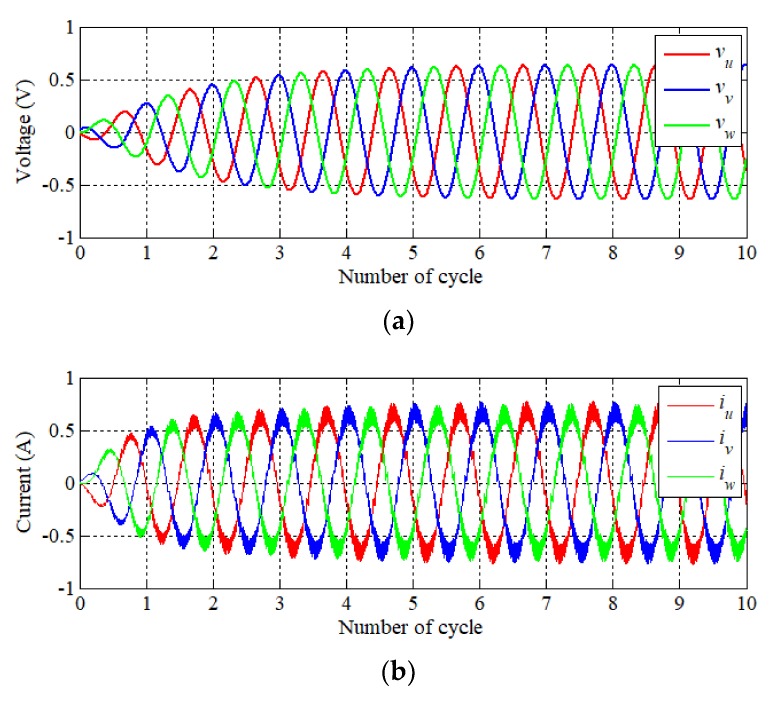

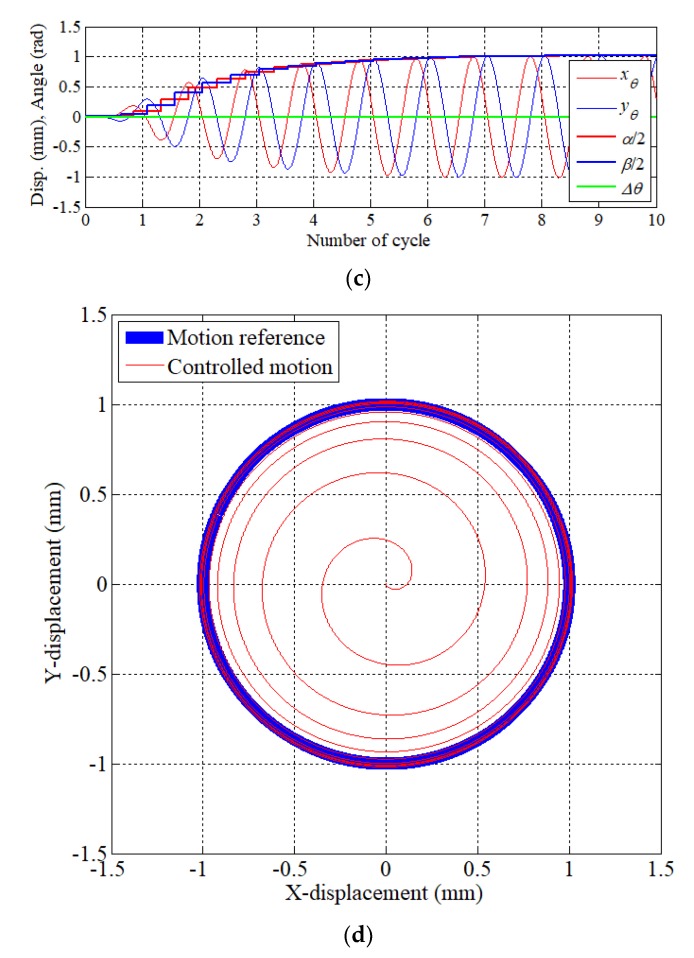
Result of scrolling motion (*α*: 2 mm, *β*: 2 mm, *θ*: 35 deg.) (**a**) Phase voltage reference, (**b**) phase current, (**c**) displacement, and (**d**) trajectory of motion.

Finally, Figure 16 presents simulation results of scrolling motion with a resonant operation. Reference stroke and angle were inputted into the controller as *α* 2 mm and *β* 2 mm. It indicates that the phase voltage and current waveforms are similar to them of three-phase PMSM. Namely, scrolling motion can be performed by applying a phase voltage of the same magnitude with a phase difference of 120°. However, a stroke of 2-DOF LRA must be controlled due to the characteristic of the unconstrained mover. Figure 16c shows that the steady-state is made within a period of six cycles. Moreover, it is controlled by the stroke of the reference value. In Figure 16d, the trajectory of the displacement on the X–Y plane is shown. It presents that resonance operation can be performed as the scrolling trajectory.

## 6. Conclusions

In this study, the authors proposed an axial gap type 2-DOF LRA which resonates with detent force without any mechanical springs. The proposed 2-DOF LRA showed that constant detent force is generated with respect to the moving direction from the FEA and simulation results. Furthermore, a method of estimating the motion of an actuator without a sensor has been proposed. Finally, it has been confirmed that various resonant motion can be precisely controlled by feedback control of the estimated values through the dynamic simulation. This study presented theoretical and analytical results. The actual design error and assembly error were not considered. Also, since the dynamic behavior of LRAs is usually unstable, these errors may make the overall system unstable. As future work, we will build a prototype to experimentally verify the effectiveness of the proposed method.

## Figures and Tables

**Table 1:** Simulation condition.

Parameter	Symbol	Value	Unit
Mass of mover	*M*	60	(g)
Detent force	*F_d_*	**Look-up table**	(N)
Friction load	*F_l_*	0.5	(N)
Viscous load	*c_l_*	5.0	(Ns/m)
Force constant	*K_f_*	**Look-up table**	(N/A)
Phase resistance	*R*	0.2	(Ω)
Phase inductance	*L*	1.2	(mH)
Resonant frequency	*f_n_*	37~40	(Hz)
Operating frequency	*f_o_*	40	(Hz)
Carrier frequency	*f_c_*	960	(Hz)
DC-link voltage	*V_dc_*	3.7	(V)
Rotation angle	*θ*	35	(degree)
